# Assessing the effectiveness of an app-based child unintentional injury prevention intervention for caregivers of rural Chinese preschoolers: protocol for a cluster randomized controlled trial

**DOI:** 10.1186/s12889-021-12156-y

**Published:** 2021-11-20

**Authors:** Jieyi He, Wanhui Wang, Peishan Ning, Peixia Cheng, Jie Li, Ming Zheng, Shujuan Yuan, Lei Yang, Youyou Wu, Huiying Zong, David C. Schwebel, Yang Yang, Guoqing Hu

**Affiliations:** 1 Department of Epidemiology and Health Statistics, Hunan Provincial Key Laboratory of Clinical Epidemiology, Xiangya School of Public Health, Central South University, Changsha, 410078 China; 2 Department of Psychology, University of Alabama at Birmingham, Birmingham, AL USA; 3 Department of Biostatistics, College of Public Health and Health Professions, Emerging Pathogens Institute, University of Florida, Gainesville, FL USA; 4 National Clinical Research Center for Geriatric Disorders, Xiangya Hospital, Central South University, Changsha, China

**Keywords:** Unintentional injury, Rural preschooler, Cluster randomized controlled trial, Application (app), Mobile health, Intervention

## Abstract

**Background:**

Compared to urban children, children living in rural areas of most countries, including China, are at higher risk of suffering unintentional injuries. Most proven injury prevention interventions, however, are rarely implemented in rural China due to lack of resources. Mobile health interventions are low-cost and easy-to-implement, facilitating implementing injury prevention in resource-limited areas (e.g., rural areas). This study is designed and implemented to examine the effectiveness of an app-based intervention for unintentional injury prevention among rural preschoolers in China.

**Methods:**

A single-blind, 18-month, parallel-group cluster randomized controlled trial with 1:1 allocation ratio will be implemented in 2 rural areas of China (Yang County, Shaanxi Province, and Shicheng County, Jiangxi Province). In total, at least 3508 rural caregivers of preschoolers aged 3–6 years old who own a smartphone will be recruited from 24 preschools. Clusters will be randomized at the preschool level and allocated to the control group (receiving routine school-based education plus app-based parenting education excluding unintentional injury prevention) or the intervention group (receiving routine school-based education plus app-based parenting education including unintentional injury prevention). External support strategies will be adopted by local partners to minimize user fatigue, non-compliance, and attrition. Data collection will be conducted at baseline and then every 3 months during the 18-month follow-up time period. Intention-to-treat data analysis will be implemented. Missing values will be imputed by using the Expectation Maximization algorithm. Generalized estimating equation will test the overall effectiveness of the app-based intervention. A per-protocol sensitivity analysis will be conducted to test the robustness of results. Subgroup analyses will follow the strategies for primary analyses. The primary outcome measure is the incidence rate of unintentional injury among preschoolers during the study period. Secondary outcome measures comprise longitudinal changes in caregiver’s attitudes, caregiver-reported supervision behaviors, and caregiver-assessed home environment safety surrounding child unintentional injury prevention in the last week using a standardized audit instrument.

**Discussion:**

The app-based intervention is expected to be feasible and effective over the 18-month intervention period. If the app is demonstrated effective as hypothesized, we will initiate processes to generalize and popularize it broadly to rural child caregivers across China.

**Trial registration:**

ChiCTR2000037606, registered on August 29, 2020.

## Background

Child unintentional injury continues to be a significant public health challenge in many countries worldwide, including China [1]. According to the Global Burden of Disease (GBD) study group 2019, approximately 38,000 Chinese children aged 0–14 years died from unintentional injury in 2019, and an additional 10,520,000 experienced unintentional injury events [2].

Various evidence-based child injury prevention interventions have been recommended by agencies such as the World Health Organization (WHO) and the United Nations International Children’s Emergency Fund (UNICEF), such as use of child restraints and seat-belts, child helmets, child-resistant containers, child safety locks, and smoke alarms [3]. However, these interventions are not consistently implemented in China, perhaps in part because no national agency has been assigned to coordinate national injury prevention efforts and promote the use of recommended interventions [4–6].

For a wide range of inequalities – including huge gaps in citizen safety awareness, adult supervision, injury prevention efforts, safety-related behavior patterns, health care services, as well as imbalanced and inadequate social-economic development between urban areas and rural areas [7–10] – children living in rural areas of China are significantly more likely to suffer an injury event than children living in urban areas consequently[11, 12]. The latest Chinese Disease Surveillance Points (DSP) data show that 2019 injury mortality rates in rural areas were significantly higher than in urban areas for under-1 children (21.56 vs. 14.55 per 100,000 population), children aged 1–4 years old (14.59 vs. 10.45 per 100,000 population), and children aged 5–14 years old (9.81 vs. 6.95 per 100,000 population) [13].

Traditional injury prevention programs require significant human, material, and financial resources to implement. Those resources are unavailable or limited in rural China. Mobile health (mHealth) interventions offer a solution that thoroughly overcomes many barriers of traditional interventions and have tremendous potential to deliver evidence-based interventions to rural citizens timely, broadly, conveniently, and efficiently, greatly boosting the accessibility and availability of health care services [14].

Successful delivery of mHealth interventions does require the recipients to have smartphones. According to *the 47th China Statistical Report on Internet Development* [15], the number of rural internet users has increased rapidly in China over the last decade, reaching 309 million by December 2020. Stated differently, internet penetration in rural areas of China has reached 55.9% and is anticipated to continue to grow rapidly [15]. This trend supports design and implementation of mHealth intervention programs for rural Chinese populations, including efforts to prevent child unintentional injury in rural areas of China.

Many mHealth interventions have been developed already for chronic disease management, maternal and infant health promotion, infectious disease prevention and control, and health services delivery [16–18], but our extensive literature searches find just 8 randomized controlled trials targeting child unintentional injury prevention via mHealth strategies [19–26]. This includes 4 trials in the United States, 1 in Australia, and 3 in China. All the eight programs were designed for urban children and implemented an intervention time interval of 8 months or less. Furthermore, seven trials used knowledge, attitudes, or behaviors as primary outcome measures to evaluate effectiveness rather than actual injury events. The only trial that examined actual changes in injury incidence rate used a 6-month intervention period and did not demonstrate the significant effectiveness [24]. User fatigue and resultant non-compliance to the intervention, plus substantial attrition from the study, were interpreted as reasons that the intervention was ineffective [27]. So far, the long-term effectiveness of mobile health technology in reducing actual child injury incidents has not been assessed in any published study.

We overcome all these limitations in the previously-proposed research. Specifically, we have refined and reinforced an app-based intervention that was previously designed and evaluated by our research team for unintentional injury prevention among caregivers of preschool children in urban Chinese areas [24]. The refinements overcome deficiencies revealed in the previous trial and meet specific needs of rural children and caregivers based on rural environment, injury spectrum of rural children, and characteristics of rural child caregivers comprehensively [28]. In this proposed research, the optimized app-based intervention will be conducted, and the sample monitored, for 18 months. We will use actual injury events as the primary outcome measure as well as considering participants’ attitudes, supervision behavior, and home environment safety. Sufficient external support from local cooperative partners will be implemented to rigorously minimize non-compliance and attrition. This study is the first mHealth randomized controlled trial (RCT) for caregivers of rural Chinese preschoolers to demonstrate the long-term effectiveness of preventing child unintentional injury via an app-based intervention and we hypothesize the innovative app-based intervention for 18 months will be effective in reducing child unintentional injury risk in rural China.

## Methods

### Study design

A single-blind, 18-month follow-up, parallel-group cluster randomized controlled trial with 1:1 allocation ratio will be implemented in Yang County, Shaanxi Province, and Shicheng County, Jiangxi Province, China. This trial was registered on August 29, 2020 in the Chinese Clinical Trial Registry (https://www.chictr.org.cn/showproj.aspx?proj=60036, registration number: ChiCTR2000037606).

### Sample size

Based on previous literature [29], we conservatively hypothesize that the unintentional injury incidence of rural preschoolers aged 3–6 years old in project sites is 30% in the past 18 months. On the basis of a previous report [30], we assume the effect size of the app intervention to be 0.80 (incidence rate ratio) compared to traditional unintentional injury prevention intervention. In addition, we assume an intra-class correlation (ICC) of 0.005 with a cluster size of 150 children per preschool [31], and an allocation ratio of 1:1 between intervention and control groups. Considering extensive strategies will be adopted to increase retention in the study, we assume an attrition rate of 15% for the 18-month follow-up. Given these assumptions, a minimum sample size of 3508 caregivers is needed for the study to achieve a statistical power of 80% at the 0.05 significance level. As many rural preschools have more than 150 children in the study sites we select, 24 preschools will be ample to recruit the needed sample size.

### Preschool recruitment

We will recruit participants from 24 preschools in China, 14 in Yang County, Shaanxi Province and 10 in Shicheng County, Jiangxi Province. Only preschools with more than 150 enrolled students will be eligible for this study; almost all preschools in the study sites are this size or larger, therefore the exclusion criteria for preschool size will have minimal impact on generalizability of results.

**Fig. 1:**
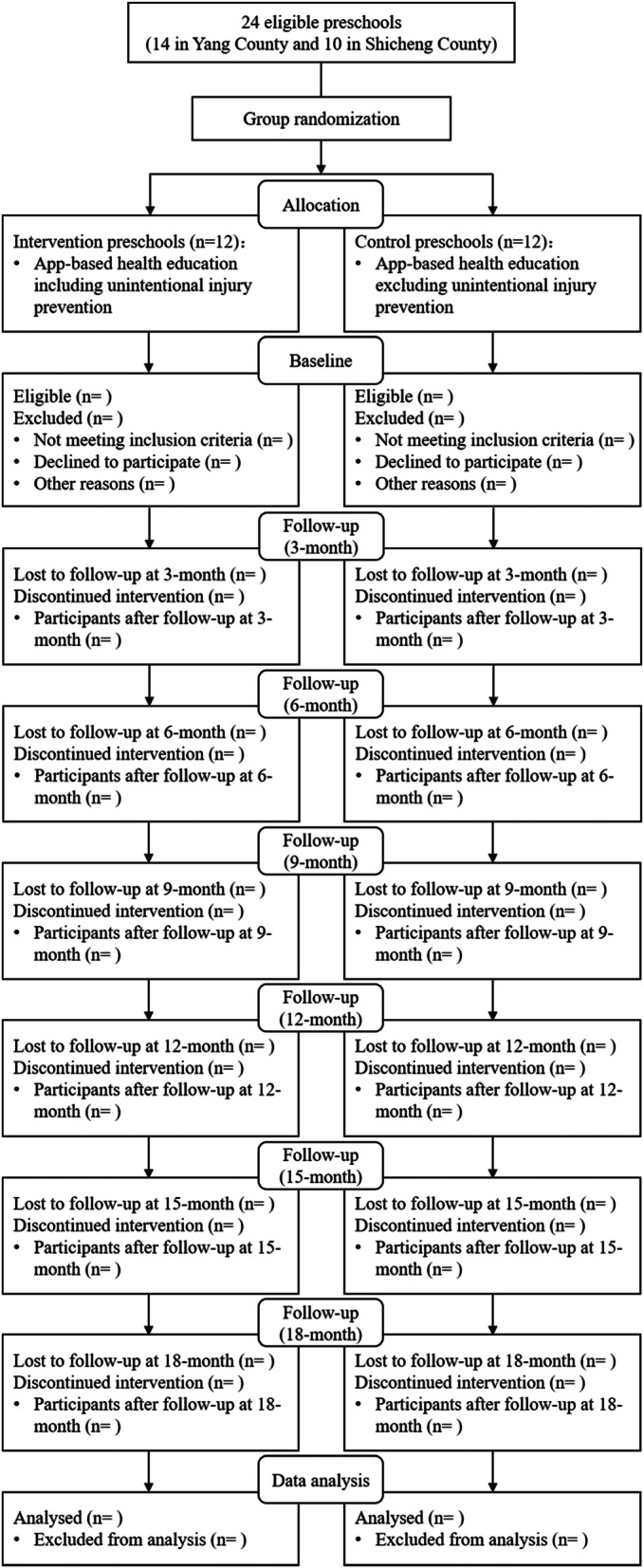
Flow diagram of selection of study participants

**Fig. 2:**
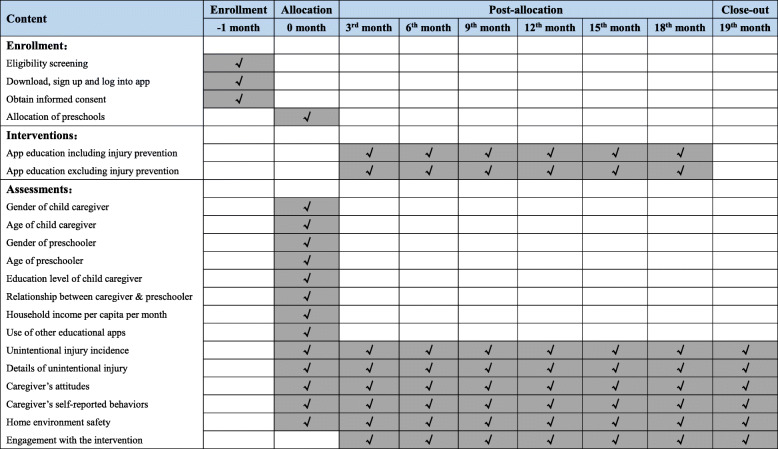
Timeline for the schedule of enrollment, interventions, and assessments

Each invited preschool will receive an official invitation letter along with relevant materials concerning the study. We will randomly allocate enrolled preschools rather than classes or individuals to the intervention group versus control group to avoid undesirable contamination within the same preschool. Thus, 12 preschools will be randomly allocated to the intervention group and 12 to the control group (Figs. 1 and 2).

### Participant recruitment

Eligible participants meet the criteria: being the primary caregiver of a preschooler attending the included schools.

Potential participants will be excluded from this study if they meet any of the following criteria: (1) unwilling to participate in this study; (2) do not own a smartphone or cannot operate the app independently; (3) cannot read text messages; or (4) expect to move to another city with their children during the study time period. If an eligible caregiver care for more than one eligible preschooler, we will enroll only the youngest child.

In coordination with local cooperative partners (governmental departments and agencies), we will recruit class teachers and school staffs to help coordinate recruitment of study participants in each selected preschool. Those individuals will inform eligible caregivers about the study via multiple existing preschool-family communication channels, such as social media platforms (WeChat or QQ), school apps, printed briefing materials and oral notification. Caregivers who agree to participate will be provided an invitation letter that includes the benefits and responsibilities of participating in this study and instructions to download, install, sign up, log in and use the randomly-assigned app. Moreover, all caregivers who agree to participate will receive a short online guidance video that detailedly introduces basic information about the study and educates the participants on how to utilize the app.

When participants log into the app for the first time, they will be invited to complete and submit the informed consent documentation online. All consenting participants will then complete the baseline survey, which collects information about unintentional injuries the child experienced in the past 3 months as well as information on socio-demographic characteristics, caregiver’s attitudes, caregiver-reported behaviors surrounding child unintentional injury prevention, and caregiver-reported home environment safety based on the standardized safety checklist over the prior week.

### Randomization and blinding

Once recruited, each enrolled preschool will be randomly allocated to a group according to the order of computer-generated random number sequence by an independent (masked) researcher. Group allocation will be concealed during data analysis.

### Interventions

Both the intervention and control groups will receive routine school-based education from each included preschool plus the app-based education programs. Routine school-based education encompasses health education, including injury prevention, that is performed by preschool teachers. This education is typically based on the suggested course from the Ministry of Education of China and influenced somewhat by the teacher’s knowledge and preferences for teaching injury prevention to children.

The basic framework for child unintentional injury prevention in the app is based on previous theories and research [32]. It comprises four active modules: (1) content learning, including a series of instructional materials to impart basic and practical knowledge and skills; (2) social interaction, consisting of an online forum, online expert consultation opportunities, and locations for users to leave comments that facilitate communication among users and between users and experts; (3) survey and feedback, used for online survey data collection and user’s suggestions, and for experts to reply to user queries; and (4) a personalizing module, allowing users to customize the color scheme of their app and to display the user’s personal learning progress. The four active modules will be identical for both groups during the program and are detailed along with the app design and other functions in a previous publication [32].

The intervention will differ between the intervention and control groups based on the learning contents they receive. The control group will receive a parenting health education program covering topics like pediatric disease risks, childhood nutrition, early childhood development, and child health care. It will exclude any explicit information about unintentional child injury prevention. The intervention group will receive all contents the control group receives plus additional components focused on prevention of common child unintentional injuries in rural areas (e.g., road traffic injury, falls, drowning, suffocation, poisoning, burning, scalding, electric current burns, and other injuries caused by animate or inanimate mechanical forces).

A previous report evaluated a precursor version of this app that was created for urban caregivers [24]. The app has been optimized and refined substantially for this trial to focus on the characteristics and needs of children and families in rural China and to overcome some deficiencies identified in the previous trial. The following specific changes were made:

1. Enriched and updated the scenarios of knowledge segments tailored to common situations in rural areas of China;
2. increased number and diversified categories of audio recordings (30% vs. 0% of contents in current vs. previous app version), interactive games (20% vs. 3%), cartoons (10% vs. 5%), and videos (10% vs. 6%);
3. reduced proportion of short essays with pictures (30% vs. 86%), based on user’s feedback;
4. increased frequency of dissemination of knowledge segments from 4 times a week to 7 times a week for both groups;
5. introduction of new learning approaches (e.g., audio learning and regular online theme activities via the app forum); and
6. improved user experience and motivation through upgraded strategies for this app, such as additional online video instruction for using the app, automatic display and interaction with the banner and recommended readings in the app’s homepage, refining online expert consultation and customer service, publishing comments and displaying other users’ comments, and changing the font color in the forum to reduce eye strain.

In addition, the time period of this intervention study will be prolonged for 18 months to demonstrate its long-term effectiveness in reducing injury incidence rate. The previous trial for urban families lasted just 6 months.

All components of the app will be available throughout the study period, ensuring that all users in both groups can retrieve and learn from knowledge segments freely and repeatedly. Except for the differences in the content of the app, the implementation of the app-based intervention will be identical for both groups.

### Strategies to enhance engagement

In order to enhance engagement with the app-based intervention, we will adopt an external support strategy. Specifically, with the support of local partners including the local education bureau, health committee, maternal and child health hospital, centers for disease control and prevention, a coordinating group will be established for each enrolled preschool. The coordinating group that is composed of local office staff members of the charitable foundation “World Vision”, class teachers, and school staffs of each enrolled preschool, will help study participants solve problems with app use and encourage engagement in the study.

Besides sufficient external support from the coordinating groups, several other strategies will be flexibly implemented to encourage active use of the app and participation in the study. First, in both intervention and control groups, a small amount of app-based reward points will be granted based on the participant’s engagement. The reward points will be exchanged for small gifts such as gift cards to refill mobile phone and data services. Second, participants who log in the app for 7 consecutive days will gain a chance to enter a lottery and win additional reward points. Third, we will provide a small gift to students monthly if their caregivers rank among the top 30 active learners over the past month. Finally, automated reminders will be sent to caregivers if they do not read app-delivered knowledge segments or fail to accomplish online questionnaires. If automated reminders fail, the coordinating group will take other actions to remind the participants such as sending a text or voice message or making a phone call.

### Outcome measure

The primary outcome measure will be the incidence rate of unintentional injury among preschoolers in the past 18 months, which will be calculated by combining data from the six follow-up evaluations accumulatively. We will conduct follow-up evaluations on a quarterly basis (every 3 months) to reduce recall bias in participants remembering children’s minor or moderate injuries [24].

An injury event will be included if it meets any of three criteria [24]: (1) child receives a medical diagnosis or treatment by a doctor or other medical professional following an injury; (2) child receives first aid, takes any injury-related medication, or receives massage or cold/hot compress by a family member, teacher or other non-medical staff following an injury; or (3) child is restricted from school or other activities, or is kept in bed or rest for more than a half-day following an injury. If a caregiver reports that a child experienced more than one unintentional injury event in the past 3 months, we will record the number of injury events but collect specific information only about the most serious one.

Unintentional injury incidence rate will be calculated as “number of preschoolers newly experiencing unintentional injury events divided by total number of preschoolers×100%”.

We also will consider several secondary outcome measures, including: (1) the caregiver’s attitudes toward child unintentional injury prevention, (2) caregiver-reported supervision behaviors related to child unintentional injury prevention in the last week, and (3) the caregiver-reported home environment safety based on a standardized audit checklist over the last week. All secondary outcome measures are based on previously-published and validated items [24, 33–36]. Table 1 lists all planned survey items, with the final survey questionnaire to be refined through a pilot survey, as detailed below.

**Table 1:** Survey items assessing child caregiver’s attitudes, supervising behaviors, and child home environment safety

**1. Caregiver’s attitudes** (with four response options: completely agree, partly agree, not sure, and completely disagree)	
a. Preventability of preschooler unintentional injury	
b. Self-efficacy to keep child safe from unintentional injury	
c. Necessity of preventing unintentional injury	
**2. Caregiver’s supervising behavior** (with five response options: always, often, seldom, never, and not applicable)	
**2.1 Risky behaviors**	
a. Letting child cross the road alone	
b. Taking child to cross a road at a location without a pedestrian crossing	
c. Letting child swim in a pond or river without adult supervision	
d. Looking left and right before taking child to cross the road	
e. Letting child ride a bicycle or scooter on the road	
f. Holding the child or letting the child sit alone on the front passenger seat when riding in a car	
g. Leaving child playing alone on the bed or stairs	
h. Leaving the child alone for a short time when bathing	
i. Adding hot water when the child is still in the bathtub	
j. Feeding child while the child is playing, laughing, or crying	
k. Giving child a large piece of food, or a whole round food (e.g., grape, nut)	
l. Leaving child at home alone	
m. Leaving the child unattended while the adult is cooking or doing housework	
**2.2 Safe behaviors**	
a. Using child restraints for child when taking a car	
b. Requiring child to wear safety protective equipment when riding a bicycle or scooter	
c. Testing water temperature before bathing the child	
d. Pouring the water out of the bathtub immediately after using it	
e. Keeping hot substances and lighters out of child’s reach	
f. Keeping sharp objects out of child’s reach	
g. Keeping medicines, detergents, sanitizers, antiseptics, and pesticides out of child’s reach	
h. Keeping small objects out of child’s reach	
**3. Home environment safety** (with six response options: completely like this, mostly like this, partly like this, mostly unlike this, completely unlike this, and not applicable)	
**3.1 Risky home environment items**	
a. Tablecloths or cloth coverings on tables and cabinets can be pulled	
b. Power cords for electric appliances are not fixed	
c. There are tables, chairs stools, or cabinets beside balconies or windows	
d. There are water spills or oil stains on the floor	
e. There are toys or dolls in the child’s bed	
f. Kitchen supplies like pots, bowls, plates, and cups are placed in child’s reach	
g. Plastic bags or cling wraps are placed in child’s reach	
h. Large round foods like grapes or nuts are placed in child’s reach	
i. Chemicals are stored in non-original containers	
**3.2 Safe home environment items**	
a. There are protectors on sharp corners	
b. Switches of water dispensers and gas stoves are equipped with protective devices	
c. Balconies and windows are equipped with guard railings	
d. Child’s bed is equipped with guard railings	
e. There are soft protective materials on the floor beside the child’s bed	
f. Water storage supplies are lidded	
g. There are anti-skid devices in the washing and bathing places	

### Pilot testing

Prior to commencing the formal study, we will recruit 60 caregivers of preschoolers to participate in feasibility testing (30 from Yang County and 30 from Shicheng County). During a one-week pilot testing phase, participants will complete two online questionnaires. One will collect complete data listed in Table 1 as well as socio-demographic factors, and the other will assess the user’s experience concerning the contents, readability, functions, interface, operability, and usability of the app. The results of feasibility testing will be used to finalize the survey questionnaire and make final refinements to the app.

### Collection of formal data

Survey data will be collected at baseline and every 3 months during the entire 18-month follow-up period. All data will be collected online through the app and stored in a password-protected backend database. If caregivers fail to complete an app-based online survey, designated members of the coordinating group will remind or guide them. When needed, a phone-based survey interview will be employed.

Participant engagement data will be gathered and stored in the backend database automatically through embedded tracking of app usage. Data to be collected include: (1) the frequency of logins, (2) time span from login to logout each time, (3) frequency of reading instructional materials, (4) time spent on learning instructional materials, (5) number of reading or bookmarking knowledge disseminations, (6) number of posting comments on knowledge disseminations, (7) number of likes clicked on knowledge disseminations, (8) frequency of joining a discussion in the online forum, (9) number of bookmarked online forum themes, (10) number of comments posted in the online forum, and (11) number of likes clicked in the online forum.

### Data analysis plan

Primary data analysis will follow an intention-to-treat (ITT) approach. Descriptive statistics will be calculated for socio-demographic variables and primary and secondary outcomes, including mean (or median) and standard deviation (or range and interquartile range) for continuous variables, and frequency and proportion for categorical variables. For each time point, Chi-square and two-sample *t*-tests (or Wilcoxon rank sum test if data are skewed) will be used to examine the differences in outcome measures between intervention and control groups for categorical and continuous variables, respectively. Trend tests or analysis of variance (ANOVA) will used to detect within-group differences across the six follow-up visits.

Generalized Estimating Equation (GEE) will be used to assess the overall effectiveness of the app-based intervention, while adjusting for socio-demographic variables, engagement, and baseline injury events.

A Generalized Linear Mixed Model (GLMM) will be used to verify the GEE results. With GLMM, missing values can be imputed using the Expectation Maximization (EM) algorithm. A per-protocol (PP) sensitivity analysis will be conducted to validate ITT results. All statistical analyses will be performed using R version 4.0.4. All statistical tests will be two-sided at the significance level of 0.05.

Subgroup analyses will be conducted to assess the impact of socio-demographic factors on the intervention effect, including gender and age of children and their caregivers, type of children’s caregivers (e.g., parents, grandparents, or others), education level of caregivers, and household income per capita per month. Subgroup analyses will employ the same statistical methods used for the primary analyses.

This study will strictly adhere to the Consolidated Standards of Reporting Trials (CONSORT) 2010 statement [37] and the Standard Protocol Items: Recommendations for Interventional Trials (SPIRIT) 2013 statement [38] to analyze the data and report the results.

## Discussion

Compared with traditional intervention strategies, mHealth technologies are cost-effective and efficient to disseminate health and safety information and to deliver health care services [39, 40]. As the number of smartphones grows worldwide, the advantage of mHealth programs has become increasingly evident [41, 42]. The potential for mHealth programs is particularly valuable to reach remote and rural populations, greatly promoting the equalization and universalization of public health services [43].

Our research uses the power of mHealth to reach rural Chinese families and teach caregivers about child unintentional injury prevention, adding evidence on the effectiveness of mHealth intervention on child unintentional injury prevention [44]. The proposed app is adopted from a previous version developed for urban child caregivers. It incorporates substantial changes based on previous experiences as well as tailoring the program to meet the needs of rural families. We propose and integrate several strategies, including use of local external support, to maintain participants engaged in using the app-based intervention program for whole 18-month intervention period.

This study is the first mHealth RCT that aims to examine the app-based child unintentional injury prevention intervention for caregivers of rural Chinese preschoolers and will adhere strictly to widely recognized guidelines for the design and conduct of randomized controlled trials. High-quality research evidence will be generated and should fill in the knowledge gaps in this field, support innovative intervention-programming and prospective policy-making, as well as further future research as anticipated. If the app proves effective as hypothesized, we will distribute to all Chinese residents without charge.

We also note that the app may have utility outside of China. We will partner with collaborators from other low- and middle-income countries to appropriately translate and adapt the intervention to local cultures and disseminate it to populations where injury prevention programming is much needed, many of them faced with markedly higher injury rates than in high-income countries [3, 45].

## Data Availability

The datasets generated and analyzed during the current study are not publicly available due to confidentiality policies, but are available from the corresponding author on reasonable request.

## Abbreviations

GBD: Global Burden of Disease
WHO: World Health Organization
UNICEF: United Nations International Children’s Emergency Fund
DSP: Disease Surveillance Points
mHealth: Mobile health
RCT: Randomized controlled trial
ICC: Intra-class correlation
ITT: Intention-to-treat
ANOVA: Analysis of variance
GEE: Generalized estimating equation
GLMM: Generalized linear mixed model
EM: Expectation maximization
PP: Per-protocol
CONSORT: Consolidated standards of reporting trials
SPIRIT: Standard protocol items: recommendations for interventional trials
